# Unveiling Fundamentals of Multi-Beam Pulsed Laser Ablation in Liquids toward Scaling up Nanoparticle Production

**DOI:** 10.3390/nano14040365

**Published:** 2024-02-16

**Authors:** Oleksandr Gatsa, Shabbir Tahir, Miroslava Flimelová, Farbod Riahi, Carlos Doñate-Buendia, Bilal Gökce, Alexander V. Bulgakov

**Affiliations:** 1 HiLASE Centre, Institute of Physics of the Czech Academy of Sciences, Za Radnicí 828, 25241 Dolní Břežany, Czech Republic; oleksandr.gatsa@gmail.com (O.G.); mirka.flimelova@hilase.cz (M.F.); 2 Chair of Materials Science and Additive Manufacturing, University of Wuppertal, Gaußstr. 20, 42119 Wuppertal, Germany; tahir@uni-wuppertal.de (S.T.); riahi@uni-wuppertal.de (F.R.); carlos.donate-buendia@uni-wuppertal.de (C.D.-B.); goekce@uni-wuppertal.de (B.G.); 3 GROC·UJI, Institute of New Imaging Technologies, Universitat Jaume I, Av. De Vicent Sos Baynat s/n, 12071 Castellón, Spain

**Keywords:** pulsed laser ablation in liquids, high-entropy alloys, diffractive optical elements, nanoparticle yield, beam splitting, cavitation bubble

## Abstract

Pulsed laser ablation in liquids (PLAL) is a versatile technique to produce high-purity colloidal nanoparticles. Despite considerable recent progress in increasing the productivity of the technique, there is still significant demand for a practical, cost-effective method for upscaling PLAL synthesis. Here we employ and unveil the fundamentals of multi-beam (MB) PLAL. The MB-PLAL upscaling approach can bypass the cavitation bubble, the main limiting factor of PLAL efficiency, by splitting the laser beam into several beams using static diffractive optical elements (DOEs). A multimetallic high-entropy alloy CrFeCoNiMn was used as a model material and the productivity of its nanoparticles in the MB-PLAL setup was investigated and compared with that in the standard single-beam PLAL. We demonstrate that the proposed multi-beam method helps to bypass the cavitation bubble both temporally (lower pulse repetition rates can be used while keeping the optimum processing fluence) and spatially (lower beam scanning speeds are needed) and thus dramatically increases the nanoparticle yield. Time-resolved imaging of the cavitation bubble was performed to correlate the observed production efficiencies with the bubble bypassing. The results suggest that nanoparticle PLAL productivity at the level of g/h can be achieved by the proposed multi-beam strategy using compact kW-class lasers and simple inexpensive scanning systems.

## 1. Introduction

Pulsed laser ablation in liquids (PLAL) is a well-established versatile technique to produce high-purity colloidal nanoparticles (NPs) of a large variety of materials [1,2,3,4]. The PLAL-produced NPs often possess superior properties as compared to other synthetic approaches, which makes them attractive for various applications. In particular, the ligand-free nature of these NPs allows for their exceptional catalytic activity [5,6] and the generation of compositionally complex NPs can be precisely controlled, thus enabling tuning of their functional properties [1,7]. For instance, PLAL has proven to be an efficient technique to produce multi-metallic nanoalloys with homogenous elemental distributions in contrast to chemical methods where it is difficult to avoid element segregation due to different redox potentials [7,8]. 

Despite the outstanding functional properties of PLAL-produced NPs, their industrial applications are still very limited. This is mainly due to the major drawback of the method, its low productivity [9]. Considerable efforts have been made to enhance the NP yield by ablation in liquid flow [10,11], optimization of the irradiation conditions [12,13,14], and variations of the target geometry [15,16,17], which allowed us to achieve productivity for various NPs (metals, dielectrics, semiconductors) at the level of ~100 mg/h, which is, however, insufficient for most applications. The main limiting factor for the PLAL efficiency is the vapor cavitation bubble generated in the target–liquid interface, which shields the subsequent laser pulses from the target [18,19]. The typical diameter and lifetime of the cavitation bubble under PLAL vary in the ranges of 0.1–1 mm and 100–300 μs, respectively, depending on irradiation conditions and ablated material [20,21,22,23]. This imposes limitations on the pulse repetition rate and does not allow one to use the full potential of modern high-power (>100 W) ultrafast (<10 ps) laser systems [9]. A milestone in the PLAL synthesis of NPs was achieved when the cavitation bubble was spatially bypassed using a high-speed (up to 500 m/s) polygon scanner, increasing the NP productivity to the industrial-scale level of several g/h [9,24]. However, the polygon scanner is an expensive and rather troublesome device that prevents its wide usage in the industry. Therefore, there is still a significant demand for a practical, cost-effective method for upscaling PLAL synthesis [14]. Examples of the fields where high productivity of NP synthesis in the multiple-gram range is of key importance include biomedical applications and heterogeneous catalysis [1,4]. Moreover, simple economic considerations show that PLAL synthesis becomes cost-effective as compared to wet chemical methods only at fairly high NP productivity [1]. 

Diffractive optical elements (DOEs) enable the reshaping of laser beams to almost any desirable distribution by diffraction on specially designed microstructured surfaces [25]. In particular, DOE beam splitters can split the incident laser beam into any number of beams along a line or in a 2D pattern and maintain other parameters of the light source (beam size, divergence, and polarization). Modern DOEs are flexible and reliable devices that can be easily incorporated into advanced optical systems and are currently widely used in applications such as laser material processing [26,27,28], optical sensing [29,30], lithography [31], and fluorescence microscopy [32]. It is reasonable to expect that multi-beam irradiation offered by DOEs can help to bypass the PLAL-generated cavitation bubble both temporally (the same ablation conditions per beam can be achieved at lower laser repetition rates) and spatially (lower beam scanning speeds are needed). Very recently, the multi-beam PLAL approach was employed for the generation of gold and iron-nickel alloy NPs and the synthesis productivity was increased 3–4 times compared to the standard single-beam setup without changing the NP properties [33].

In this paper, we investigated the effects of laser beam splitting on NP productivity by PLAL using different types of DOEs and lasers and demonstrated possibilities to bypass the cavitation bubble both spatially and temporally. As a model material, we employed a multimetallic high-entropy alloy (HEA) composed of Cr, Fe, Co, Ni, and Mn in equimolar proportions. HEAs have become one of the most popular material classes in recent decades in both fundamental and applied materials science due to their unique mechanical, electromagnetic, and chemical properties [34,35]. HEA NPs have considerable advantages over bulk materials in various functional properties and show great potential for applications such as catalysis, energy storage, bioimaging, and chemical sensing [36,37,38]. For instance, HEA NPs were shown to outperform most previously reported electrocatalysts [39]. However, the controllable and efficient synthesis of HEA NPs is still a challenge [36,38,39]. It was recently demonstrated that PLAL is a promising technique for the production of such NPs [40]. In this study, we employ the MB-PLAL scheme and analyze the NP production efficiency as a function of synthesis parameters (laser fluence, repetition rate, and beam scanning speed). We demonstrate that the proposed multi-beam method allows one to dramatically increase the PLAL productivity of colloidal NPs. Furthermore, we performed time-resolved imaging of the cavitation bubble under the considered PLAL conditions to correlate the observed increase in NP productivity with the possibility of bypassing the bubble using the proposed multi-beam strategy. 

## 2. Materials and Methods

HEA CrFeCoNiMn targets were produced by pressing micropowders (Thermo Fisher Scientific, Waltham, MA, USA) of the individual elements and oven sintering. For equimolar elemental composition, the powders of Co (99.8%, 1.6 μm), Cr (99.2%, <10 μm), Fe (99.5%, <10 μm), Mn (99.6%, <10 μm), and Ni (99.9%, 3–7 μm) were mixed in the mass ratio of 1:0.88:0.95:0.93:1. The homogenized powder mixture was pressed at 200 MPa forming 10-mm-diamter, 2-mm-thick pellets, which were sintered in an argon atmosphere at 1000 °C for 20 h. The HEA targets were deoxidized and mechanically processed to fit into the flow chamber. 

The ablation experiments were carried out in two PLAL setups. In the first setup, we used a high-power HyperRapid NXT laser (Coherent, Santa Clara, CA, USA) emitting pulses at 1064 nm, with a duration of 10 ps, a repetition rate ranging from 400 kHz to 2 MHz, and a maximal output power of 100 W. In the second setup, we employ a “middle class” 6-W Yb:KGW-based PHAROS laser from Light Conversion (Vilnius, Lithuania) operating at 1030 nm, with a pulse duration of 7 ps and repetition rates varying between 7 and 200 kHz. In both cases, the samples were positioned in identical homemade PLAL chambers of a flow-through design [11,24] with a liquid volume of 35 × 20 × 6 mm^3^ and laser-irradiated at normal incidence through a glass window. Distilled water was used as a liquid and pumped through the chamber at a flow rate of 0.125 L/min in both cases, ensuring complete filling of the chamber volume under laminar flow conditions. The thickness of the water layer between the chamber window and the target surface was 4 mm. The laser pulses were focused using f-theta lenses (f = 167 mm for 1064 nm and f = 163 mm for 1030 nm) into identical circular spots of radius *w*_o_ = 24 µm (1/e^2^ criterion). The laser pulse energy *E*_0_ was varied in the range of 10–60 µJ by either setting the output power (NXT laser) or using an attenuator consisting of a λ/2 plate and a polarizer (PHAROS laser) to achieve a peak fluence *F*_0_ = 2*E*_0_/π*w*_o_^2^ in the range 1–6 J/cm^2^. The beams were scanned over the target surface by galvanometric scanners (Raylase, SS-IV-15 1070, Wessling, Germany) for the high-power laser and ScanCube IV (Scanlab, Pichheim, Germany) for the middle-class laser with maximal scanning speeds of 20 m/s and 3 m/s, respectively. The experimental conditions used with the two laser systems are summarized in Table 1.

**Figure 1:**
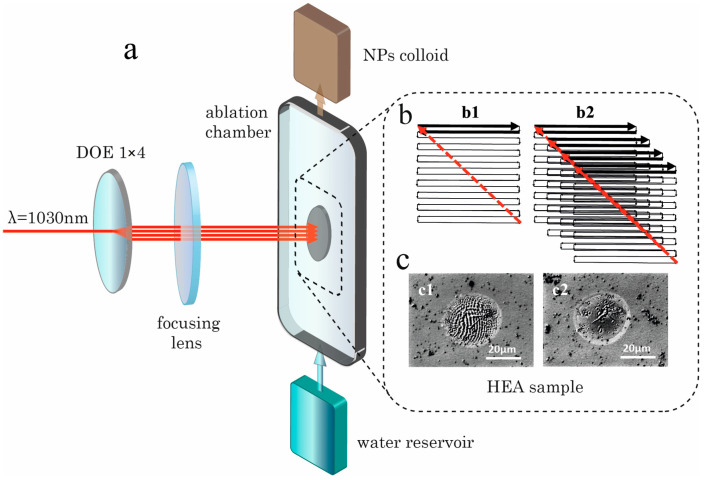
Schematic presentation of the experimental setup. (**a**) A general scheme of beam splitting and irradiation of the sample with several beams; (**b**) beam scanning pattern for a single beam (**b1**) and four beams after 1 × 4 DOE (**b2**); (**c**) optical images of spots produced at 2 J/cm^2^ by a single beam (**c1**) and one of the beams after the 1 × 4 beam splitter (**c2**).

The laser beams were split using static 1-dimensional 1 × *n* DOEs (HOLO/OR, Ness Ziona, Israel) placed in front of the galvoscanners and generating lines of *n* spots. For the NXT laser, we used a 1 × 2 DOE producing two beams while, in the experiments with the PHAROS laser, the beam was split into 4 sub-beams (1 × 4 DOE). Colloids produced under such beam-splitting conditions and the NP productivity were compared with those obtained with a single Gaussian beam (without DOEs). A general scheme of the experimental setups is shown in Figure 1a for the case of the 1 × 4 DOE configuration. A zigzag scanning pattern (4 × 4 mm^2^ area for every beam, 30 μm distance between the lines) was used (Figure 1b). The DOE-produced spot line was tilted with respect to the scanning direction to minimize the interaction of the beams with the cavitation bubbles during scanning. For the 1 × 2 DOE with its large spot separation (~9 mm), two identical HEA targets were placed in the chamber next to each other and were irradiated independently with the same zigzag scanning pattern. The DOE-generated spot on the target and a spot produced by a single original pulse at the same fluence were found to be identical in size and shape (Figure 1c). 

The productivity *M* of the PLAL synthesis of NPs (in mg/h) was determined by measuring the target mass loss during the colloid production (typically for 5–20 min depending on the irradiation conditions). For this, the target was weighed before and after the irradiation run using analytical microbalances EAB 124i (Adam Equipment, Oxford, USA) with a precision of 0.1 mg. Based on these data, the ablation rate *m* (mass removed per pulse) was also determined. The laser spots on the target surface were investigated by an optical microscope (Olympus BX43, Shinjuku, Japan). The obtained HEA NPs were analyzed by scanning electron microscopy (SEM TESCAN MIRA3 LMH, Brno, Czech Republic). 

A time-resolved shadowgraphy technique was employed to characterize the evolution of the cavitation bubble. For these experiments, the HEA target was cut in half and placed in a standard 3500 µL quartz cuvette (Thorlabs CV10Q35FA) filled with ultra-pure water (MilliQ, Millipore). The target was irradiated through the cuvette side wall by 1064-nm, 10-ps pulses from a 160-W Nd:YAG laser (PX400-3-GH, EdgeWave GmbH, Würselen, Germany) under single-shot conditions. The pulses were focused onto the HEA target surface by a f-theta lens (f = 100 mm) to a circular spot similar in size to those used in the DOE experiments. The laser pulse energy was adjusted by ND filters to the same range (10–50 μJ) as for the colloid production. The shadowgraphy images were acquired using an ICCD camera (Andor iStar DH334T-18H-13, Oxford Instruments, Oxford, UK) and a xenon flash lamp (L4633-01, Hamamatsu, Japan). High magnification was achieved using a telecentric telescope (Correctal T/1.5, Sill Optics, Wendelstein, Germany, and 4×, 65 mm CompactTL Telecentric Lens, Edmund Optics, Barrington, NJ, USA). A gate time of 300 ns was used for the image acquisition. Details of the shadography setup can be found elsewhere [41].

## 3. Results and Discussion 

### 3.1. Effect of the Pulse Repetition Rate

**Figure 2:**
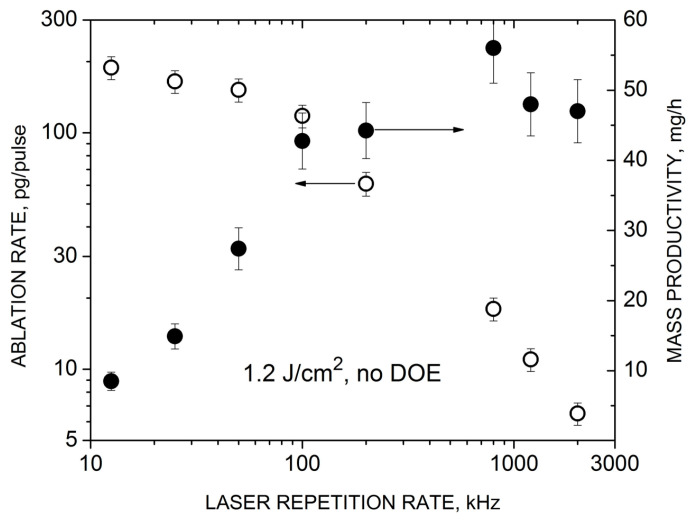
HEA ablation rate (open symbols) and NP productivity (closed symbols) under single-beam irradiation (PHAROS laser, no-DOE regime) as a function of the pulse repetition rate at fixed laser fluence (1.2 J/cm^2^) and beam scanning speed (3 m/s).

The laser repetition rate *f*_R_ is one of the key parameters determining the characteristic temporal and spatial scales of the interaction between the pulse and the cavitation bubble during PLAL, and thus the ablation efficiency and NP synthesis productivity [9,18]. We performed reference experiments on the effect of the repetition rate on the ablation rate and the total NP yield under single-beam PLAL, without beam splitting. The results for the middle-class laser for specific fluence and scanning speed values are shown in Figure 2. The error bars here and in the following figures were obtained based on 3–4 independent measurements under identical PLAL conditions from the standard error estimates. The ablation rate and productivity demonstrate opposite dependencies, and this is likely to be determined by the features of the laser–bubble interaction at different repetition rates. At low *f*_R_ values, below ~100 kHz under these PLAL conditions, and when no or little bubble-shielding effect occurs (either the bubble lifetime is lower than the interpulse time 1/*f*_R_ or the bubble size is smaller than the beam scanning distance during the 1/*f*_R_ time), the ablation rate is virtually independent on the repetition rate. Correspondingly, the productivity increases almost linearly with *f*_R_. However, at a certain value of *f*_R_, the ablation rate starts to quickly decrease, presumably due to the interaction of the laser beam with the vapor bubble generated by the previous pulse [18,19]. As a result, the NP productivity is nearly saturated in this *f*_R_ range. 

A critical repetition rate when both dependencies change their behaviors is around 100 kHz under the considered conditions (Figure 2). Based on these data, we can estimate the characteristic lifetime and size of the cavitation bubble assuming that at this repetition rate, these two bubble parameters become comparable with the interpulse time *t*_p_ and interpulse scanning distance *L*_p_. Then the bubble lifetime is ~ *t*_p_ = 1/*f*_R_ = 10 μs and its size *L*_b_ is ~ *L*_p_ *= V*/*f*_R_ = 30 μm, where *V* = 3 m/s is the scanning speed. We can consider these values as a reference when evaluating conditions for bypassing the cavitation bubble by MB-PLAL. At first glance, the obtained values appear to be too low for a ps-PLAL-generated bubble with its typical parameters reported in the literature to be in the ranges of ~100 μs and 100 μm, respectively [9,22]. However, it is important to consider that the bubble size and lifetime decrease when reducing the laser pulse energy applied [22,42]. Typically, the literature data describe relatively high pulse energies, usually above ~100 μJ, whereas the conditions in Figure 2 involve a pulse energy of approximately 10 µJ. Furthermore, the bubble is usually investigated at single-shot conditions, whereas multi-pulse irradiation, as in our case, results in smaller bubbles [43]. In Section 3.2, we demonstrate that the evaluated parameters of the cavitation bubble are quite reasonable for the considered conditions. Note that for other PLAL conditions, the critical repetition rate for bypassing the bubble can be essentially different from that found here, especially for higher pulse energies when the bubble lifetime is longer. For instance, under the ablation of zinc by 125-μJ, 7-ps laser pulses in tetrahydrofuran, the critical repetition rate was found to be only 5 kHz [18].

### 3.2. Time Evolution of the Cavitation Bubble under HEA PLAL

**Figure 3:**
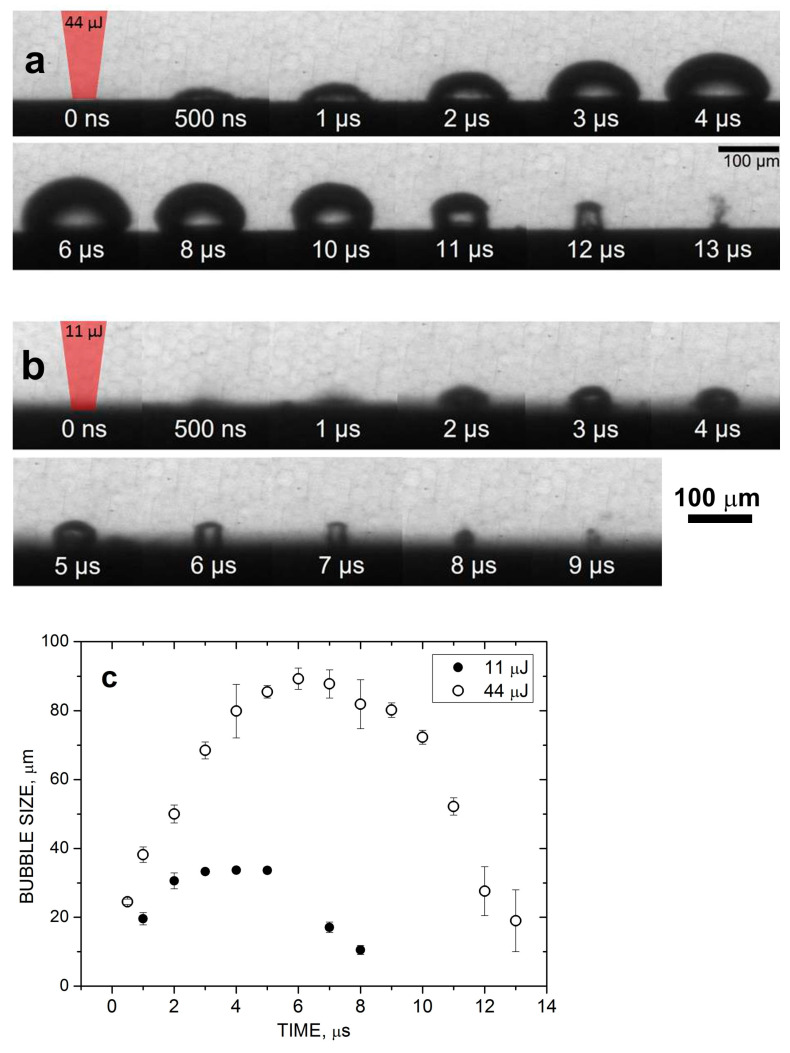
Selected shadowgraph images of the cavitation bubble produced by laser ablation of HEA in water at the laser pulse energy of 44 μJ (**a**) and 11 μJ (**b**) at different time moments. (**c**) Bubble equivalent radius as a function of time deduced from (**a**,**b**).

To reveal the cavitation bubble dynamics under the considered HEA PLAL conditions and to correlate it with the evaluated bubble parameters, we performed experiments on bubble visualization for laser pulse energies in the range of 10–50 μJ, used here for NP production. These experiments were performed under single-beam irradiation conditions, without the use of DOEs, as a reference. Figure 3 illustrates the bubble time evolution for two characteristic regimes. The first regime at *E*_0_ = 44 μJ with a fluence of ~ 4 J/cm^2^ is a typical value for PLAL NP production with the NXT laser (Figure 3a). The second regime at *E*_0_ = 11 μJ (Figure 3b, fluence *F*_0_ ≈ 1 J/cm^2^) corresponds to the conditions of Figure 2. In all cases, the bubble exhibits a semi-ellipsoidal shape before its collapse, as commonly observed under single-pulse irradiation conditions [22,43]. Figure 3c shows the temporal evolution of a bubble equivalent radius (i.e., the radius of a hemispherical bubble of the same volume as the observed oblate half-spheroid) for these regimes. Interestingly, at 11 μJ, the bubble lifetime and maximal size, ca. 10 μs and 40 μm, respectively, are in good agreement with the estimations based on the repetition rate dependences (Section 3.1). At fourfold higher pulse energy, the bubble size increases more than two times, up to ~ 90 μm, while the lifetime increases to a lesser extent, by only a factor of ~1.5 (Figure 2c). Below, we consider these values as a reference when analyzing the possibilities of bypassing the cavitation bubble using MB-PLAL.

### 3.3. Laser Fluence Effect 

To study the effect of laser fluence on the HEA ablation rate and NP productivity, we performed experiments with and without DOEs at a fixed total number of pulses *N*, irradiation time *T*, and scanning speed *V*. The repetition rate with a 1 × *n* DOE was selected as *f*_R,DOE_ = *f*_R,1_/*n* to ensure the same number of pulses at a fixed irradiation time, where *f*_R,1_ is the repetition rate in the corresponding reference regime without DOE. 

**Figure 4:**
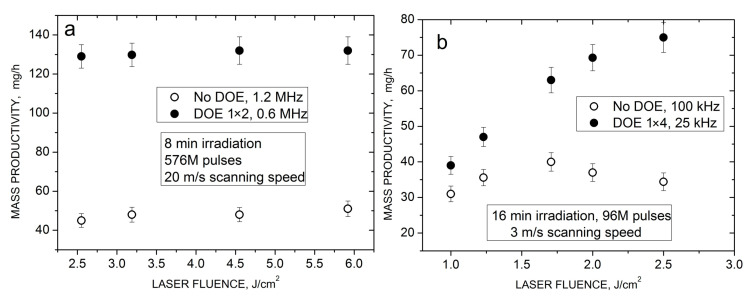
HEA NP productivity with and without DOEs as a function of laser fluence at fixed total number of pulses *N*, irradiation time *T*, and scanning speed *V*. (**a**) High-power laser (*N* = 576 × 10^6^, *T* = 8 min, *V* = 20 m/s, *f*_R_ = 1.2 MHz without DOE and 0.6 MHz with a 1 × 2 DOE). (**b**) Middle-class laser (*N* = 96 × 10^6^, *T* = 16 min, *V* = 3 m/s, *f*_R_ = 100 kHz without DOE and 25 kHz with a 1 × 4 DOE).

Figure 4a presents a comparison of the HEA NP productivity by the high-power laser with (MB-PLAL) and without DOE (PLAL) as a function of laser fluence at relatively high fluence values. Two interesting results can be noticed. First, the NP productivity does not depend on the fluence, both for PLAL and MB-PLAL. Second, the productivity with the 1 × 2 DOE is almost three times higher than that without beam splitting. It is important to note that, as the *N* and *T* values are fixed, the same fluence dependencies are valid also for the ablation rates. These observations can be plausibly explained in terms of the bubble shielding effects. At the repetition rate in the DOE regime, the interpulse time of 1.6 μs is much shorter than the bubble lifetime (~15 μs, as shown in Figure 3c) and thus the DOE cannot achieve the temporal bypass of the bubble. However, spatial bypassing appears to be relevant in this case. The interpulse distance (16 μm without DOE) is already comparable with the bubble size (Figure 3) and its two-fold increase reduces the shielding degree (refer to Section 3.4), resulting in a considerable increase in the NP yield. Nonetheless, bypassing is incomplete in these fluence regimes even for the MB-PLAL system, as the bubble size is still larger than the interpulse distance. This explains the observed weak fluence dependences. Indeed, at higher fluences, the bubble is larger and the expected increase in the ablation rate is compensated by a stronger shielding effect. Note that saturation of the ablation mass after a certain fluence was observed even under bubble bypassing conditions [9]. We believe, however, that in the considered regimes, the shielding effect is the main factor as demonstrated below with the middle-class laser.

Figure 4b shows the fluence dependencies of the NP productivity obtained with the PHAROS laser for both PLAL and MB-PLAL. These experiments were performed at considerably lower repetition rates, where not only spatial bypassing of the bubble but also its temporal bypassing plays a role. Under PLAL conditions, the *t*_p_ and *L*_p_ values are 10 μs and 30 μm, respectively, i.e., comparable with the bubble lifetime and size (Figure 3). This implies partial bypassing of the bubble in this regime, both temporally and spatially. However, for MB-PLAL, these values are 40 μs and 120 μm, much larger than the corresponding bubble parameters, indicating complete bypassing of the bubble. As a result, the fluence dependence of the NP productivity exhibits different behavior for PLAL and MB-PLAL. Without employing the DOE, the dependence is quite similar to those observed with the high-power laser (Figure 4a), namely, the productivity first slightly increases at low fluences and then saturates due to the bubble shielding effect. In contrast, with the DOE, when the shielding effect does not influence the process, laser ablation in liquids becomes comparable with ablation in air in terms of material removal [9], and the MB-PLAL productivity increases continuously with fluence in the studied *F*_0_ range (Figure 4b). This clearly demonstrates that the nearly fluence-independent ablation yield observed in Figure 4a is due to the bubble shielding effect. 

In the next two Sections, we will separately consider the effects of spatial and temporal bypassing of the cavitation bubble by MB-PLAL.

### 3.4. Spatial Bypassing of the Cavitation Bubble 

With the two different lasers, two different experimental approaches were used to isolate the effect of the interpulse distance *L*_p_ = *V*/*f*_R_. For the high-power laser, the interpulse time 1/*f*_R_ was always much shorter than the bubble lifetime in the studied *f*_R_ range, and thus irrelevant for bubble bypassing. Consequently, the distance between pulses (*L*_p_) was varied by changing the pulse repetition rate at a fixed laser fluence and scanning speed. For the middle-class laser, the 1/*f*_R_ value is comparable to or larger than the bubble size. Hence, we fixed the repetition rate and changed the scanning speed to vary the interpulse distance. 

**Figure 5:**
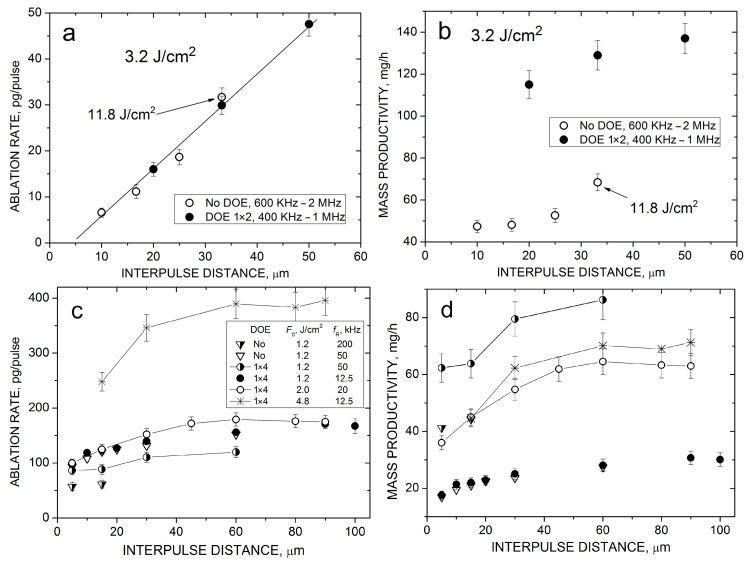
HEA ablation rate (**a**,**c**) and NP productivity (**b**,**d**) with and without DOEs as a function of the interpulse distance for PLAL with the high-power laser at a fluence of 3.2 J/cm^2^ (**a**,**b**) and with the middle-class laser at several fluences and repetition rates (**c**,**d**). The line in (**a***)* is a linear fit of the data. The lines in (**c**) and (**d**) are to guide the eye.

Figure 5 illustrates the effect of the interpulse distance on the laser ablation rate and NP productivity for the studied conditions with different beam-splitting ratios. At the higher repetition rates of the NXT laser, all the obtained productivity values follow a single linear dependence, both with and without DOE throughout the studied *V*/*f*_R_ range (Figure 5a). This means that, by increasing *L*_p_, our ~50-μm-diameter beam becomes less overlapped with the bubble produced by the preceding pulse and thus less shielded. There is no sign of saturation of the dependence which is expected to occur when the interpulse distance reaches the bubble size, i.e., at *V*/*f*_R_ ≈ 100 μm (Figure 3c), a value unachievable in these experiments due to technical limitations (*f*_R_ ≥ 400 kHz, *V* ≤ 20 m/s). A similar linear dependence was obtained for the ablation rate of metals in liquids in the *f*_R_ range of 1–10 MHz [9]. Here, the dependence is observed at repetition rates in the range of 0.4–2 MHz irrespective of whether the beam is split or not. Even a strong increase in the laser fluence from 3.2 to 11.8 J/cm^2^ almost does not affect it (Figure 5a), although the latter is rather expected due to the weak fluence dependence of the ablation mass in this regime (Figure 4a). 

Based on the obtained linear interpulse distance dependence for the ablation yield, we can estimate the benefit of using the beam-splitting technique for the PLAL NP productivity under high-repetition-rate conditions. When the beam is split into *n* sub-beams with the corresponding decrease in the repetition rate by a factor of *n* to keep the laser fluence and output power the same, both the *L*_p_ distance and ablation rate increase *n* times. Therefore, as the number of pulses in a unit of time remains unchanged, the total productivity increases by a factor of *n* (assuming that temporal bubble bypassing is of no relevance). Our measurements with the 1 × 2 DOE with the twofold increased productivity in the dual-beam regime are in good agreement with this estimation (Figure 5b).

Figure 5c,d illustrates the interpulse distance effect for the middle-class laser at relatively low repetition rates (below 100 kHz). In this scenario, the spatial bypassing of the bubble is more complicated since it is accompanied by temporal bypassing. The increase in the ablation rate with the *L*_p_ distance is weaker than in the high-repetition-rate regimes. The dependences *m*(*L*_p_) are saturated at *L*_p_~50 μm and are now fluence- and *f*_R_-dependent. Thus, when the *L*_p_ distance is enhanced from 10 to 50 μm, the ablation rate increases only by a factor of ~ 1.6, both for 2.0 and 4.8 J/cm^2^ fluences (Figure 5c), instead of the expected fivefold increase for *f*_R_ > 400 kHz. At fairly low fluences and repetition rates, the MB-PLAL does not provide any benefit in the ablation rate and NP productivity since the cavitation is already bypassed without beam splitting (comp. data for 1.2 J/cm^2^ at 50 kHz without DOE and at 12.5 kHz with DOE). However, at fourfold higher repetition rates (200 and 50 kHz, respectively) and the same laser fluence of 1.2 J/cm^2^, the 1 × 4 DOE provides a ~1.5-times increase in productivity compared to a single beam at the same laser power throughout the *L*_p_ values studied (Figure 5d). 

Note that under totally identical irradiation conditions (*F*_o_, *f*_R_, *L*_p_), the ablation rate with MB-PLAL is slightly lower than those of single-beam PLAL (comp. the *m* data for 50 kHz at 1.2 J/cm^2^, Figure 5c). This is likely due to additional beam shielding by the long-lived persistent microbubbles [14], an effect that appears to be more probable in the splitting regime. However, this reduction in the ablation rate is more than compensated by the increase in the number of pulses resulting in the enhancement of the NP productivity by a factor of ~3 under these conditions with the 1 × 4 DOE (Figure 5d). Note that the interaction of the simultaneously produced bubbles in the MB-PLAL regimes can be neglected since the individual sub-beams are well separated on the target [44,45].

### 3.5. Temporal Bypassing of the Cavitation Bubble 

**Figure 6:**
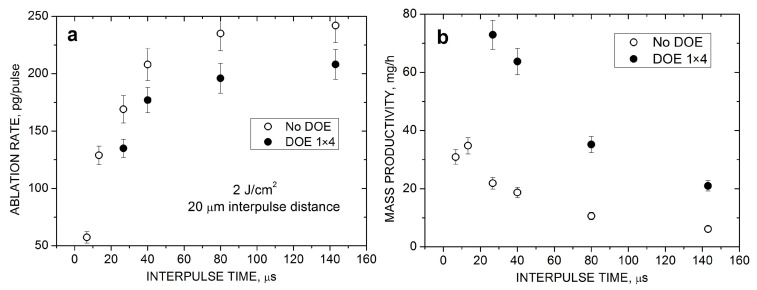
HEA ablation rate (**a**) and NP productivity (**b**) with and without a 1 × 4 DOE as a function of the interpulse time for PLAL with the middle-class laser at *F*_0_ = 2 J/cm^2^ and *L*_p_ = 20 µm.

The temporal bypassing effect was investigated for the middle-class laser at moderate pulse repetition rates. The interpulse time *t*_p_ was varied by changing the *f*_R_ value while the interpulse distance *V*/*f*_R_ was fixed by the corresponding adjustment of the scanning speed *V*. Figure 6 shows the influence of the interpulse time on the HEA ablation rate and NP productivity at a fixed laser fluence and interpulse distance. The *t*_p_ dependence for the ablation rate *m* is considerably stronger than the *L*_p_ dependence under these conditions, especially for low *t*_p_ values (comp. Figure 5c and Figure 6a). Thus, when the interpulse time increases from 6.7 μs (150 kHz) to 40 μs (25 kHz), the ablation rate increases by a factor of ~4.2 (Figure 6a), i.e., almost as strong as with the interpulse distance in high-repetition-rate regimes (Figure 5a). The *m*(*t*_p_) dependence starts to saturate at *t*_p_ ~50 μs, a value that is longer than the measured bubble lifetime of ~15 μs (Figure 3c). This is possibly due to persistent microbubbles generated after the collapse of the cavitation bubble resulting in additional beam shielding [14]. Therefore, temporal bubble bypassing appears to be a dominant factor for the enhancement of the PLAL synthesis productivity when the interpulse time becomes comparable with the bubble lifetime due to the pulse repetition rate reduction in MB-PLAL. Again, as for the *m*(*L*_p_) dependence (Figure 5c), the ablation rate for the individual sub-beams is slightly lower than that for the original laser beam under identical irradiation conditions (Figure 6a), which is, however, a minor effect compared to the total increase in the NP productivity due to the splitting method (Figure 6b). 

### 3.6. Nanoparticle Production in the Beam Splitting Regime

**Figure 7:**
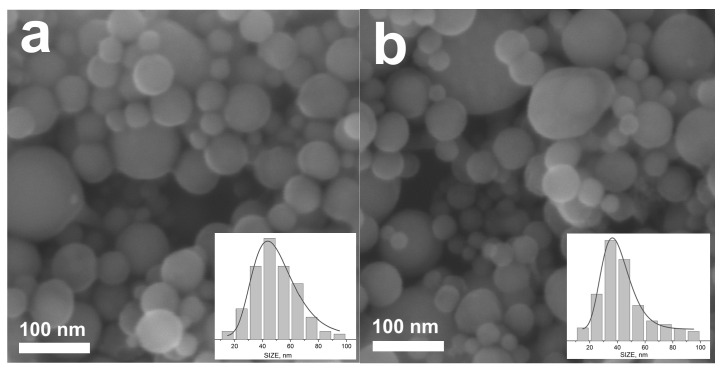
SEM images of nanoparticles produced by HEA PLAL with the middle-class laser at a fluence of 2.5 J/cm^2^ and scanning speed of 3 m/s with a 1 × 4 DOE ((**a**), *f*_R_ = 25 kHz) and without DOE ((**b**), *f*_R_ = 100 kHz). The insets show the number-weighted particle size distributions approximated by a log-log function. The mean particle size and distribution width (FWHM) are, respectively, 45 and 34 nm for (**a**) and 39 and 28 nm for (**b**).

To check whether applying the beam-splitting technique affects the properties of the synthesized nanoparticles or not, we compared HEA NPs produced by the middle-class laser in the single-pulse and multi-pulse regimes at the same total number of pulses applied. Figure 7 shows SEM images of NPs obtained with and without a 1 × 4 DOE at a laser fluence of 2.5 J/cm^2^ and employing the same output laser power when the NP productivity in the multi-pulse regime is approximately 2 times higher (Figure 4b). The corresponding particle size distributions are also shown in Figure 7. As we can see, MB-PLAL does not practically affect the shape and size of the NPs. In both cases, they are nearly spherical, have almost the same mean Feret diameters of 45 nm without DOE and 39 nm with DOE, and have similar size distributions. Although the mean size of particles obtained in the MB-PLAL regime is slightly smaller and their distribution is slightly narrower than those for single-beam-produced NPs (Figure 7), we believe that this difference is statistically insignificant, even when a fairly large number of NPs (3400) were analyzed. Note that in both regimes, we do not observe large particles with sizes in the micrometer range, which justifies the use of the gravimetric measurements of the ablation mass for evaluation of the NP yield.

## 4. Conclusions 

In this paper, we address the challenge of nanoparticle (NP) productivity by PLAL through the incorporation of static diffractive optical elements (DOEs) into the PLAL set-ups to split the laser beam into several identical sub-beams (MB-PLAL). With a multimetallic CrFeCoNiMn high-entropy alloy as a model material and with two picosecond laser systems, we investigated the beam-splitting effect on the PLAL and MB-PLAL production of NPs as a function of synthesis parameters (laser fluence, pulse repetition rate, beam scanning speed, and number of sub-beams). 

We demonstrate that the proposed multi-beam method facilitates the temporal and spatial bypassing of the generated cavitation bubbles. The MB-PLAL system is shown to achieve identical ablation conditions at lower repetition rates, increasing the interpulse distance, and hence, spatially lowering the beam scanning speeds required to reduce cavitation bubble shielding effects). This enables a significant increase in the PLAL productivity of HEA colloidal NPs. Furthermore, we performed time-resolved imaging of the cavitation bubble under the considered PLAL conditions to correlate the observed increase in NP productivity with the possibility of bypassing the bubble using the multi-beam strategy. The proposed method is universally applicable to PLAL production of NPs across a large variety of materials.

The obtained results suggest that through MB-PLAL, NP synthesis at the level of g/h can be achieved using modern compact kW-class picosecond lasers [28,46] and simple inexpensive scanning systems. Thus, considering a 200-kW, 100-kHz laser [28], its output beam can be split into ~50 sub-beams (e.g., using a standard 7 × 7 DOE) to have a pulse energy of ~40 μJ in every beam. With a typical 50-μm-diameter focusing spot, this will result in a peak laser fluence of ~4 J/cm^2^ that is sufficient for strong ablation of most materials. Using a conventional galvoscanner with a scanning speed of 3 m/s, it is reasonable to expect an ablation rate in the range of 150–200 pg/pulse for such conditions (Figure 5c). With 50 beams at 100 kHz, this would lead to NP productivity of ~3 g/h, although, of course, such extrapolations should be made with care. 

## Figures and Tables

**Table 1:** Parameters of the lasers used and ranges of the irradiation conditions.

PLAL Set-Up	High-Power Laser	Middle Class Laser
Laser	HyperRapid NXT	PHAROS Yb:KGW
Wavelength, nm	1064	1030
Pulse duration, ps	10	7
Max output power, W	100	6
Pulse energy, µJ	20–60	9–44
Peak fluence, J/cm^2^	2.5–6	1–4.8
Effective spot radius, µm	24	24
Repetition rate, kHz	400–2000	7–200
Scanning speed, m/s	20	0.0625–3
DOE used	1 × 2	1 × 4
Beam separation angle, deg	3.67	0.28
Spot separation, mm	9	0.8

## Data Availability

Data are contained within the article.
